# Critical appraisal of studies evaluating prevalence of attention deficit hyperactivity disorder

**DOI:** 10.3389/fpsyt.2025.1646618

**Published:** 2025-10-01

**Authors:** Michelle Tivadar, Sara Popit, Igor Locatelli, Matej Stuhec

**Affiliations:** ^1^ University of Ljubljana, Faculty of Pharmacy, Ljubljana, Slovenia; ^2^ Lekarna Novak, Novo mesto, Slovenia; ^3^ Pomurske lekarne, Murska Sobota, Slovenia; ^4^ Department of Clinical Pharmacy & Pharmacology, Faculty of Medicine Maribor, University of Maribor, Maribor, Slovenia; ^5^ Department of Clinical Pharmacy, Ormoz Psychiatric Hospital, Ormoz, Slovenia

**Keywords:** attention deficit hyperactivity disorder, epidemiology, prevalence, systematic review, methodological quality, critical appraisal tool

## Abstract

**Introduction:**

Attention-deficit hyperactivity disorder (ADHD) is a neurodevelopmental disorder affecting an estimated 5–7% of children and adolescents and 2–5% of adults. However, prevalence rates in published studies vary widely, largely due to methodological differences. High-quality, accurate, prevalence data are essential for clinical decision-making and policymaking. However, these data have not been consistently documented in previous meta-analyses and systematic reviews.

**Aim:**

To assess the methodological quality of studies reporting ADHD prevalence using the relevant critical appraisal tool.

**Methods:**

Our previously published systematic review identified 103 studies reporting clinically confirmed ADHD prevalence. The studies were grouped by type and age of subjects, and 101 studies were evaluated for risk of bias using an adapted Joanna Briggs Institute (JBI) critical appraisal tool modelled on the Cochrane Risk of Bias-2 (RoB2) method.

**Results:**

The Joanna Briggs Institute (JBI) critical appraisal tool was found to be the most suitable for evaluating prevalence studies. Of the studies reviewed, 62 (61.4%) were at high risk of bias, and only seven (6.9%) had a low risk. Although one- and two-stage clinical study designs are of a higher quality, they are still often highly susceptible to bias.

**Conclusion:**

The methodological quality of most ADHD prevalence studies is low. Systematic reviews must include critical appraisal to ensure the reliability of synthesised data. Accurate prevalence estimates are urgently needed in order to improve our understanding of the disease burden and enhance patient management.

## 1 Introduction

Attention-deficit/hyperactivity disorder (ADHD) is a neurodevelopmental disorder characterised by developmentally inappropriate levels of inattention, hyperactivity, and/or impulsivity (1–3). Although ADHD is commonly diagnosed in childhood, it often continues into adolescence and adulthood, and can persist throughout a person’s life (2, 4, 5). According to the World Federation of ADHD International Consensus Statement, which provided 208 empirically supported statements about the disorder, ADHD prevalence in children and adolescents ranges from 5.9% to 14%, and from 0.2% to 2.8% in adults (5, 6).

Several systematic reviews and meta-analyses have explored the global prevalence of ADHD, particularly among children and adolescents. Reported estimates have varied widely – from as low as 1% to nearly 20% in school-age populations (7). These substantial variations are primarily attributable to differences in methodological procedures, including diagnostic criteria, study design, sampling strategies, and geographic and cultural contexts (5, 8–10).

A further source of heterogeneity in ADHD prevalence studies lies in the approach to diagnosis and study design. Prevalence estimates may be based either on clinical diagnoses, where a qualified professional (e.g. a psychologist, psychiatrist, or other healthcare provider) formally confirms the disorder, or on non-clinical assessments, such as self- or parent-reported questionnaires. Additionally, studies vary in whether they use registry and administrative data (e.g., medical records or national health databases) or survey-based methods (e.g. asking participants if they have ever been told by a doctor that they have ADHD). These differences affect the validity and comparability of prevalence estimates.

Reliable prevalence estimates are essential for health-related planning, resource allocation, and evidence-based policymaking (11). Nevertheless, the credibility of these reviews depends significantly on the methodological quality of the included studies and the transparency of risk of bias assessment (11, 12). Poor study design and inadequate reporting can introduce bias into the findings and lead to inaccurate conclusions, thereby undermining the evidence base (12).

Upon reviewing the literature, we observed that many systematic reviews and meta-analyses on ADHD prevalence did not consistently report on the quality of the included studies or provide a critical appraisal. Furthermore, in certain instances, risk of bias was assessed using methodologically inappropriate approaches (e.g., utilizing STROBE as a quality assessment instrument rather than a reporting guideline). A significant proportion of systematic reviews on global ADHD prevalence have not evaluated the methodological quality of the included studies, which limits the comparability of the results and contributes to the substantial heterogeneity in prevalence estimates. This inconsistency impairs the comparability of findings across reviews and may contribute to the substantial heterogeneity observed in prevalence estimates. Key sources of heterogeneity include differences in diagnostic frameworks (e.g., DSM vs. ICD), age groups, geographic regions, sampling methods, and response rates. A summary of the systematic reviews and the quality assessment tools they used is provided in the **Supplementary Material** (see **Supplementary Table S1**). Most older reviews – particularly those published before 2015 – did not apply formal risk-of-bias assessment tools. Early reviews such as those by Scahill et al. (13), Polanczyk et al. (2007 and 2014) (8, 9), Simon et al. (14), Willcutt et al. (15), and Catalá-López et al. (16), either did not assess quality or only addressed it briefly in the limitations discussion.

In contrast, more recent studies have increasingly incorporated structured and transparent methods to evaluate study quality. A number of validated tools are currently available to facilitate the assessment of bias in prevalence studies nd their use has become more common in recent years. Migliavaca et al. conducted a review of these tools, noting substantial variability in their structure and assessed domains (11). Among the available tools, the Joanna Briggs Institute (JBI) Prevalence Critical Appraisal Tool is widely regarded as the most methodologically robust for the evaluation of prevalence studies (11, 17, 18). Other instruments frequently utilized in this context include the Risk of Bias Tool for Pre valence Studies by Hoy et al., the Newcastle–Ottawa Scale (NOS), the STROBE statement (predominantly a reporting guideline), and AMSTAR 2 for the evaluation of systematic reviews (19–23). For example, Thomas et al. (24), Wang et al. (25), and Reale et al. (26) applied modified versions of the Hoy tool, while Dobrosavljevic et al. (27), Cénat et al. (28), Lynch et al. (29), Jakobsson et al. (30), and Azmeraw et al. (31) used JBI-based checklists. Umbrella reviews and large-scale meta-analyses published since 2020, such as those by Ayano et al. (5, 32), and Sacco et al. (33), more consistently applied formal risk-of-bias evaluations using AMSTAR or AXIS.

In view of the considerable uncertainty regarding the quality of bias assessment in many systemic reviews and meta-analyses on the prevalence of ADHD, there is an evident necessity for research that prioritizes the reliability and validity of these data. Accurate burden estimation, informed policy, and effective service planning all depend on high-quality prevalence research. In response, we developed and adapted a critical appraisal tool specifically for assessing the methodological quality of ADHD prevalence studies. Our aims were: (1) to establish a transparent and rigorous tool tailored to clinically defined ADHD prevalence studies, and (2) to apply this tool to a selection of primary studies identified our previous paper (34). Our goal was to identify common sources of bias and contribute to a more robust framework for evaluating ADHD prevalence among children, adolescents, and adults.

## 2 Methods

### 2.1 Search strategy and inclusion criteria

The searching strategy has already been published in a meta-analysis in our previous paper (34). In brief: A comprehensive PubMed/MEDLINE search was performed up to January 2, 2024, including studies in all languages. Eligible studies were observational cohort (retrospective, prospective, or registry-based), cross-sectional, and clinical studies from general population samples where ADHD was diagnosed either clinically (per ICD or DSM criteria) or through validated research scales. Only studies involving cases of ADHD that had been clinically diagnosed by a psychiatrist, psychologist, paediatrician or other qualified medical professional specialising in psychiatry, and that had been performed in a clinical setting, including a clinical interview conducted by a psychiatrist in combination with other diagnostic tools (e.g. screening questionnaires) or on their own, were considered. Studies in which ADHD diagnoses were recorded in health databases, as well as surveys in which participants reported having received an ADHD diagnosis from a qualified physician, were also included. However, studies identifying ADHD cases based solely on screening tools completed by parents or teachers were excluded. Participants included in the selected studies were stratified by age group. The studies were classified into four categories: one-stage and two-stage clinical studies; and survey- and registry-based studies utilising medical records data.

### 2.2 Selection of a tool for the quality assessment

We primarily employed the Joanna Briggs Institute (JBI) critical appraisal tool, which is designed for use in systematic reviews, to critically evaluate the included studies and assess the reliability and relevance of prevalence studies (19). This checklist evaluates the risk of bias in a study’s design, conduct, and analysis. It consists of nine questions, each with four possible responses: “yes,” “no,” “unclear,” or “not applicable”.

### 2.3 Quality assessment process and adaptation of the quality assessment tool

The developers of the JBI tool emphasise that the decision to include or exclude a study from a systematic review ultimately rests with the reviewer (19). To enhance the objectivity and validity of our assessments, we employed a hierarchical evaluation approach for overall risk of bias, inspired by the Cochrane RoB 2 tool (35, 36).

#### 2.3.1 Adaptation of the JBI checklist for prevalence studies: grouping and assessment process

The Joanna Briggs Institute (JBI) Critical Appraisal Tool for Prevalence Studies is a widely used and validated instrument designed to evaluate the methodological quality of observational studies on various clinical topics. However, its general structure, intended for broad applicability, can limit its precision when applied to specific research areas, such as ADHD prevalence. To address this, we adapted and reorganised the original nine JBI items into four conceptual domains (D1–D4), each representing a critical area where bias may be introduced in prevalence studies. This domain-based structure enables a more systematic and transparent assessment, particularly suited to the diverse methodological demands of ADHD prevalence research. Study design differences—such as clinical vs. population-based samples, diagnostic procedures, or sampling strategies—necessitate tailored appraisal criteria. Our upgraded tool reflects these needs by providing a refined framework aligned with the unique characteristics of ADHD prevalence studies. The four domains and their corresponding items are described in detail below.

D1. Risk of bias in sample selection:

This domain includes questions that assess the representativeness and adequacy of the sample:

- Question 1 from the JBI checklist: Representativeness of the sample frame
- Question 2 from the JBI checklist: Sampling method
- Question 3 from the JBI checklist: Sample size
- Question 4 from the JBI checklist: Characteristics of the participants and the research environment

D2. Risk of bias in measurement and classification:

This domain focuses on the accuracy and reliability of the measurement tools used in the study:

- Question 6 from the JBI checklist: Validity of the methods used
- Question 7 from the JBI checklist: Standardization and reliability of the measurements

D3. Risk of bias due to non-response:

This domain evaluates potential bias introduced by non-respondents or attrition:

- Question 5 from the JBI checklist: Adequacy of sample coverage in the analysis
- Question 9 from the JBI checklist: Appropriateness of the response rate

D4. Risk of bias in statistical analysis:

This domain addresses the robustness and appropriateness of the statistical methods used:

- Question 8 from the JBI checklist: Statistical analysis procedures

#### 2.3.2 Evaluation process and overall assessment

For each question, we assessed the potential for bias by evaluating the quality of the reported information and the adherence to best practices for that aspect of the study design. The overall evaluation was conducted at two levels:

- Individual question assessment: Each question was rated based on the provided responses (Yes, No, Unclear, Not Applicable), with additional interpretation where necessary (e.g., “unclear” responses indicating poor or insufficient reporting).
- Domain-level assessment: As per the Cochrane Risk of Bias 2 (RoB 2) tool, each domain was assessed using a series of signalling questions (36). If the response to any signalling question within a domain indicated a high risk of bias, the entire domain was rated as such. The responses to the questions within each domain were then aggregated to form an overall risk assessment
- Overall risk rating: The domain ratings were combined into an overall judgment of each study’s risk of bias. The final overall risk was classified into three categories:
  • Low risk: If all domains were rated as low risk.
  • Some concerns: If at least one domain raised concerns (e.g., unclear responses or unclear reporting).
  • High risk: If any domain was rated as high risk.

By employing this structured approach, we aimed to enhance the assessment’s objectivity while ensuring that the final risk ratings accurately reflected the potential biases in the study design, measurement, non-response handling, and statistical analysis.

## 3 Results

### 3.1 Quality assessment process

We began our evaluation with Question 8, which focuses on statistical analysis. However, because a meta-analysis has been conducted and all statistical data have been re-analysed, including calculation of missing values such as confidence intervals and prevalence by age and gender, this domain was considered less critical for our initial assessment (37). When such data were missing, we added the comment “find additional information” and did not formally assess the domain for bias. Nonetheless, study design and statistical strength are still addressed in other tool parts. For example, Question 3 pertains to sample size calculation, which influences statistical power, while Questions 2 and 9 address proper weighting in the sampling process or adjustments for attrition affecting representativeness.

In cases where studies used a two-stage approach (e.g., initial screening followed by diagnostic confirmation) but failed to account for false negatives from the screening stage in their prevalence estimates, we considered their statistical analysis flawed. These studies were automatically rated as having a high overall risk of bias.

**Figure 1:**
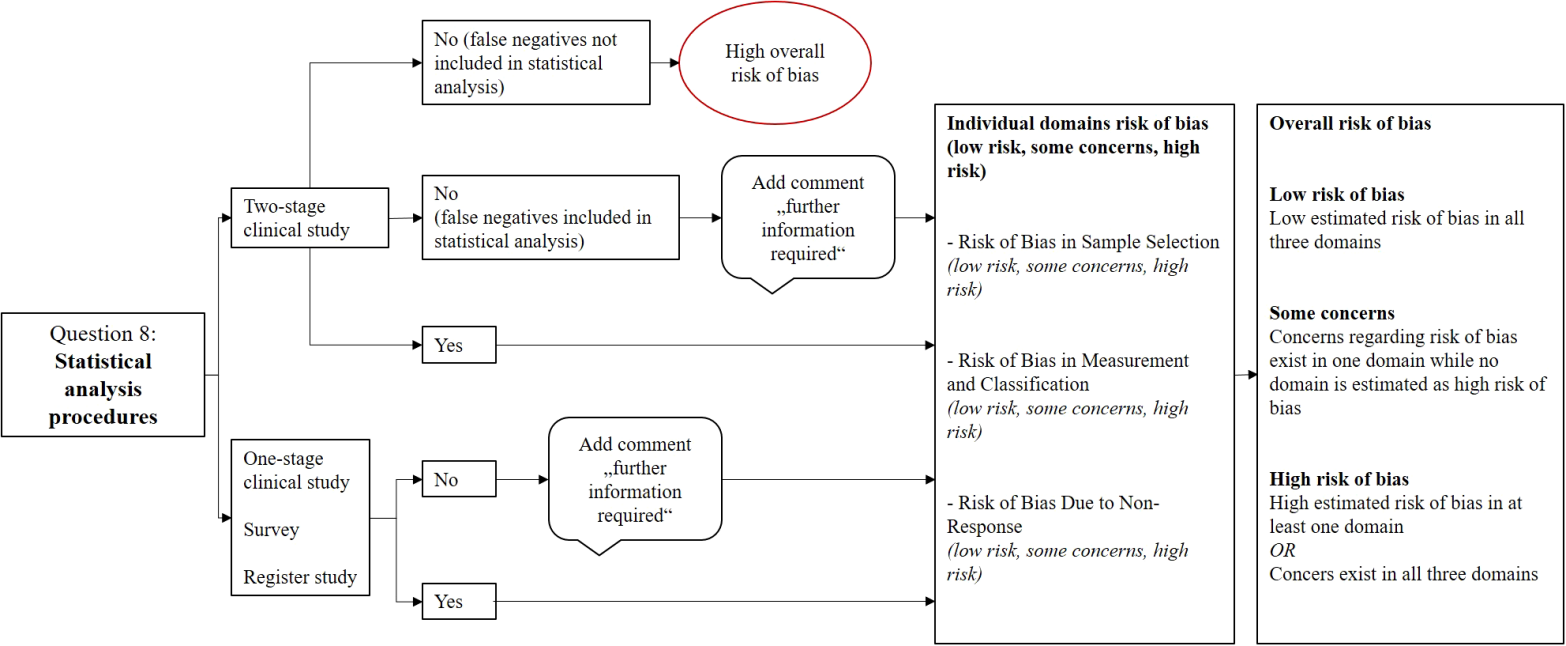
Process of critical appraisal for question 8.

We continued our evaluation domain by domain, assessing the remaining questions accordingly. The results from each domain were then integrated into a final judgment of each study’s overall risk of bias. **Figure 1** provides a visual representation of this evaluation framework.

Within the JBI checklist for studies reporting prevalence, we adapted and expanded the criteria to improve objectivity and clarity. **Table 1** presents a comparison of the Original JBI Tool vs. the Adapted JBI-based risk of Bias Assessment.

**Table 1:** Comparison of the original JBI tool and the adapted JBI-based risk of bias assessment used in this study.

Component	Original JBI tool for prevalence studies	Adapted version (this study)
Purpose	Assess methodological quality of prevalence studies for systematic reviews	Enhance objectivity and domain-specific bias evaluation in prevalence studies using a structured, hierarchical approach
Number of Questions	9 checklist items	9 checklist items (with modified interpretation and aggregation)
Response Options	Yes/No/Unclear/Not applicable	Yes/No/Unclear/Not applicable
Overall Assessment	No explicit formula; left to reviewer judgment	Hierarchical synthesis modeled after Cochrane RoB 2; overall risk of bias determined by a highest domain-specific rating
Bias Domains	No formal domain categorization	Questions grouped into 4 bias domains: D1. Risk of Bias in Sample Selection (Q1–Q4) D2. Risk of Bias in Measurement and Classification (Q6–Q7) D3. Risk of Bias Due to Non-Response (Q5, Q9) D4. Risk of Bias in Statistical Analysis (Q8)
Special Handling of Questions	All items are treated equally	Q4 excluded from the algorithm due to reliance on reporting quality Q8 de-emphasized due to independent meta-analysis and re-analysis of data
Interpretation of Poor Reporting	“Unclear” or “No” responses treated conservatively (Hoy et al.)	Introduced “unclear” to reflect poor or ambiguous reporting; distinguished between non-reporting and non-compliance
Handling of Two-Stage Studies	Not explicitly addressed	Studies failing to account for false negatives in two-stage designs are automatically rated high risk in the statistical domain
Response Rate Evaluation	Lower response rates are considered potentially problematic	Response rate <50% not automatically considered high risk; adjusted if sample was weighted or >300 with no bias evidence; negligible for registry studies
Thresholds for Domain Ratings	Not defined	Risk per domain:• Low: clear criteria met• Some concerns: unclear/incomplete info• High: criteria unmet or flawed methodology
Overall Risk Levels	Reviewer discretion	Structured into 3 tiers:• Low Risk• Some Concerns• High Risk (based on highest domain rating)
Use of External Assumptions	Not specified	1:1 gender ratio assumed if not reported, but only if the sample was randomly drawn from the general population
Influence of Sample Size	Not emphasized	Small sample size considered a critical issue; underpowered studies rated high risk
Documentation and Transparency	Checklist-based	The algorithm and decision rules are outlined in the **Supplementary Material** for transparency and reproducibility

### 3.2 Evaluation of the selected studies

We evaluated 101 studies (38–138) reporting the prevalence of ADHD, which were identified in our previous paper (34), using the modified JBI critical appraisal tool. Of these, 46 were two-stage clinical studies, 24 were one-stage clinical studies, 8 were survey-based studies, and 23 studies estimated prevalence using data from medical databases.

In summary, only 7 studies (6.9%) were rated as having a low overall risk of bias. Of these, 5 were two-stage clinical studies—including one focused on preschool children and children, one on adults, and one on children—while two were a single-stage clinical studies involving children and adolescents. This corresponds to 10.9% of two-stage clinical studies and 8.3% of one-stage clinical studies being assessed as low risk regarding potential bias affecting prevalence estimates.

In contrast, 32 studies (31.7%) were assessed as having some concerns, and 62 studies (61.4%) were rated as having a high risk of bias.

Of the studies, 94 (93.1%) used valid diagnostic methods for ADHD, while seven (6.9%) used methods that were unclear. Nevertheless, only 55 studies (54.5%) were assessed as having a low risk of bias in the measurement domain, while six studies (5.9%) were rated as high risk in this domain.

In the domain of sample selection, 42 studies (41.6%) were found to be at high risk of bias, while 36 studies (35.6%) were rated as low risk. Regarding the non-response domain, where applicable, 30 studies (29.7%) were assessed as low risk, and 19 studies (18.8%) as high risk.

When analysing by age groups:

- Preschool children were included in 21 studies (exclusively or alongside other age groups). Of these,
  • 9 studies (42.9%) were rated as high risk,
  • 1 study (4.8%) was low risk, and
  • 11 studies (52.4%) were considered to have some concerns.
- Children were the focus of 69 studies (either exclusively or in combination). Of these,
  • 42 studies (60.9%) were rated high risk,
  • 5 studies (7.2%) were of low risk, and
  • 22 studies (31.9%) were identified as having some concerns.
- Adolescents were included in 58 studies. Of these,
  • 35 studies (60.3%) were rated high risk,
  • 2 studies (3.4%) as low risk, and
  • 21 studies (36.2%) were considered to have some concerns.
- Adults were included in 11 studies (either alone or with other age groups). Of these,
  • 6 studies (54.5%) were rated high risk,
  • 2 studies (18.2%) were of low risk, and
  • 3 studies (27.3%) were considered to have some concerns.
- All age groups were included in 7 studies. Of these,
  • 6 studies (85.7%) were rated high risk, and
  • 1 study (14.3%) was considered to have some concerns.

## 4 Discussion

This study makes two key contributions to ADHD epidemiology: (1) a critical evaluation of existing prevalence studies using clinical diagnostic criteria, and (2) the development and application of an adapted version of the Joanna Briggs Institute (JBI) critical appraisal tool for prevalence studies. To our knowledge, this is the first time that such a modified JBI framework has been applied systematically and in such detail to ADHD research. Our findings offer a pathway toward more standardised and rigorous critical appraisal practices in ADHD prevalence research and prevalence studies more broadly.

In this study, we applied critical appraisal to the 101 studies that defined the prevalence of clinically confirmed ADHD and were included in our 2024 meta-analysis (see the **Supplementary Material**, **Supplementary Table S2**) (34). We enhanced the appraisal to reflect the stricter quality standards established through our adapted appraisal tool. We have previously published a systematic review in the European Psychiatry (34) using the PRISMA approach, which is recommended for systematic reviews. However, the PRISMA approach is not very specific in terms of critical appraisal. Therefore, we decided to address this issue in a separate paper that includes the JBI tool. Through this study, we developed an adapted, domain-based critical appraisal tool that addresses methodological features specific to ADHD prevalence research. Another reason is associated with study types. PRISMA mainly focuses on clinical studies, which are not necessarily applicable for registry-based studies. In this context, we modified the JBI tool. We hope that this modified tool will make critical appraisal in ADHD epidemiological studies more accurate.

The adapted appraisal tool addresses several limitations inherent in the original JBI checklist, most notably the absence of a validated scoring system and the reliance on subjective judgement. In light of the inconsistencies across existing risk of bias tools, we developed a hierarchical evaluation scheme to facilitate more objective assessments. Our approach involved conducting a comparative analysis of the original JBI tool, RoB 2, and a JBI-based tool that had been adapted specifically for the context of our study. This structured approach allows for more meaningful comparisons between studies and provides researchers conducting systematic reviews and meta-analyses in similar fields with a practical resource.

To address the limitations of item-level interpretation in the original checklist, we organised the nine JBI items into four key domains—each representing a potential pathway through which bias may be introduced in observational prevalence studies. The rationale for aggregating domain-level assessments into a single overall risk of bias rating—categorised as *low*, *some concerns*, or *high*—is grounded in the need to summarise complex evaluations in a manner that facilitates interpretation, comparison, and synthesis, This approach aligns with the practice adopted in several established tools (e.g., RoB 2, GRADE) that balance detail with usability. Overall ratings were derived through a transparent, rule-based synthesis of domain-level findings. The domain structure ensures that the complexity of each study’s methodological strengths and weaknesses is preserved throughout the appraisal process, while the final summary risk rating serves as a practical means for interpreting and integrating findings into meta-analyses and broader reviews. Our approach represents a step toward more standardised, structured, and replicable critical appraisal practices in ADHD epidemiology—and potentially beyond.

Our analysis focused exclusively on studies reporting prevalence based on clinically confirmed ADHD diagnoses, to ensure higher diagnostic validity. When developing our evaluation strategy, we deliberately avoided the simplified scoring system used in previous reviews [e.g., Dobrosavljević (27); Cénat et al. (28)], which gives equal weight to all checklist items. This can mask significant methodological flaws, meaning that studies with poor sampling or invalid measurement methods are rated similarly to those with only minor flaws. Our findings demonstrate the importance of a more nuanced assessment framework.

A key finding of this review is that 61.4% of studies that used clinical methods to estimate ADHD prevalence were rated as being at high risk of bias. Only a small number met the criteria for low risk. Had we excluded all high-risk studies, more than half of the literature would have been removed from the analysis. This highlights a critical tension in prevalence research: while many studies contain methodological weaknesses, they may still offer valuable insights, particularly in populations or settings that have not been extensively researched. Therefore, the results of systematic reviews should be interpreted with careful consideration of study quality, rather than relying on binary inclusion criteria.

We also observed considerable heterogeneity in the results of studies with different designs, particularly in two-stage clinical evaluations. This underscores the necessity of future systematic reviews and meta-analyses that account for variability in study design when estimating global ADHD prevalence. The modified tool developed in this study provides a framework for such work and could form the basis of improved critical appraisal practices in psychiatric epidemiology. Although PRISMA tools are widely used in this process, they are not specific enough, and an upgrade may be required to guide researchers more effectively in assessing study quality. Our results suggest that there is a need for more refined tools to better guide researchers in assessing and minimising bias in prevalence research. To enhance objectivity and minimise bias in the selection and appraisal of studies, risk-of-bias assessments should be conducted by at least two independent reviewers, with a third reviewer resolving any disagreements (11, 12, 139). Systematic review authors are encouraged to transparently present quality assessments in both narrative summaries and structured tables, highlighting any concerns, methodological inconsistencies, or noteworthy findings.

Despite its strengths, this study has several limitations. Firstly, the evaluation tool used was more complex and time-consuming than the JBI approach, as it required strict criteria for assigning a low risk of bias. Consequently, only a few studies received such ratings. This may have created the misleading impression that the overall quality of studies in this field is poor. Secondly, we did not distinguish between fully structured and semi-structured diagnostic interviews, which could affect prevalence estimates. Similarly, we did not analyse the influence of different informants (e.g., parents or teachers), although including clinical assessments likely reduced related bias. Thirdly, the poor quality of reporting in many studies limited our ability to accurately assess the risk of bias. Thirdly, the poor quality of reporting in many of the studies meant that we were unable to accurately assess the risk of bias. In several cases, we were unable to confirm or rule out potential sources of bias due to a lack of information. For example, Froehlich et al. (82), received a higher risk rating due to a lack of detail regarding who conducted the clinical interviews. This emphasises the importance of adhering to established reporting guidelines, such as the STROBE statement. Moving forward, authors may also benefit from using structured tools, such as the JBI checklist that was applied in our review, to ensure more transparent and comprehensive reporting. Fourthly, although domains marked as “not applicable” were not rated—as bias could not logically arise in those cases—the design of certain studies, particularly survey- and registry-based ones, still posed challenges for risk of bias assessment. For instance, in survey studies reporting clinician-diagnosed ADHD and in analyses using health registry data, question 7 of the JBI checklist frequently impeded a judgment of low risk of bias. This was due to uncertainty about whether the diagnosis was applied consistently across all participants, as variability in diagnostic procedures and judgment between different clinicians is likely. Studies in which a single clinician or clinical team assessed all participants were considered more reliable in this regard. Although the relevance of question 7 in these cases is debatable, its inclusion highlights a key limitation—yet it may also give a misleading impression that the overall study quality is poor. In the case of studies that utilized administrative databases, three of the nine checklist questions were found to be inapplicable. This underscores the necessity for a risk of bias instrument that is customized for such designs, one that also considers other relevant sources of bias, such as the utilization of validated case definitions to mitigate the risk of including false positives in the final analysis.

As part of our appraisal, we excluded two studies from our systematic review. Bishry et al. (140) relied primarily on screening tool data (CASS:S) to report ADHD prevalence, however the diagnostic confirmation using K-SADS-PL revealed that only 12 out of 87 screened children met the criteria. This indicates that the reported prevalence was not based on clinical evaluation alone. Anderson et al. (141) were excluded due to their use of outdated diagnostic criteria that were inconsistent with contemporary DSM/ICD standards. We also highlight the study by Bannett et al. (47), which derived ADHD prevalence estimates from electronic health records, but which did not aim to assess prevalence. Therefore, it was assessed as being at high risk of bias, primarily due to issues with the sampling frame.

Our findings emphasize the importance of rigorous critical appraisal in ADHD prevalence reviews and meta-analyses. Authors should report their methods and procedures with greater precision to enable accurate assessment of study quality. The field would benefit from the wider adoption of structured and transparent evaluation frameworks to improve the comparability and validity of studies. These frameworks would support more accurate prevalence estimates and inform evidence-based planning in mental health services and research. Researchers should consider using enhanced appraisal tools when planning studies to proactively minimise bias, particularly during the design and registration phases of clinical and epidemiological research.

## Data Availability

The original contributions presented in the study are included in the article/**Supplementary Material**. Further inquiries can be directed to the corresponding author.

## Supplementary material

Supplementary Table 1 Summary of systematic reviews and corresponding study quality assessment tools.

Supplementary Table 2 Assessment of the methodological quality of ADHD prevalence studies using the adapted JBI tool.
