# Supplementary material for: Critical appraisal of studies evaluating prevalence of attention deficit hyperactivity disorder
Source: Front Psychiatry. 2025 Oct 1;16:1646618. doi: 10.3389/fpsyt.2025.1646618 (PMC12521103; doi:10.3389/fpsyt.2025.1646618)
Supplement: Supplementary Table 1 — Summary of systematic reviews and corresponding study quality assessment tools. [file Table1.docx]

Supplementary Material

# Supplementary Figures and Tables

Table S1. Summary of Systematic Reviews and Corresponding Study Quality Assessment Tools.

| **Author (year of publication)** | **Title** | **Quality assessement** |
| --- | --- | --- |
| Scahill et al. (2000) (1) | Epidemiology of ADHD in school-age children | n/a |
| Skounti et al. (2007) (2) | Variations in prevalence of attention deficit hyperactivity disorder worldwide | n/a |
| Polanczyk et al. (2007) (3) | The worldwide prevalence of ADHD: a systematic review and metaregression analysis | n/a |
| Simon et al. (2009) (4) | Prevalence and correlates of adult attention-deficit hyperactivity disorder: meta-analysis | n/a |
| Farah et al. (2009) (5) | ADHD in the Arab world: a review of epidemiologic studies | n/a |
| Shooshtary et al. (2010) (6) | The prevalence of Attention Deficit Hyperactivity Disorder in Iran: A systematic review | “Quality Assessment Checklist for Prevalence Studies” |
| Willcutt et al. (2012) (7) | The prevalence of DSM-IV attention-deficit/hyperactivity disorder: a meta-analytic review | n/a |
| Catalá-López et al. (2012) (8) | Prevalence of attention deficit hyperactivity disorder among children and adolescents in Spain: a systematic review and meta-analysis of epidemiological studies | n/a |
| Bakare et al. (2012) (9) | Attention deficit hyperactivity symptoms and disorder (ADHD) among African children: a review of epidemiology and co-morbidities | n/a |
| Ramos-Quiroga et al. (2013) (10) | Attention deficit hyperactivity disorder in the European adult population: prevalence, disease awareness, and treatment guidelines | n/a |
| Polanczyk et al. (2014) (11) | ADHD prevalence estimates across three decades: an updated systematic review and meta-regression analysis | n/a |
| Polanczyk et al. (2015) (12) | Annual research review: A meta-analysis of the worldwide prevalence of mental disorders in children and adolescents | n/a |
| Thomas et al. (2015) (13) | Prevalence of attention-deficit/hyperactivity disorder: a systematic review and meta-analysis | modified tool developed by Hoy et al. |
| Alhraiwil et al. (2015) (14) | Systematic review of the epidemiology of attention deficit hyperactivity disorder in Arab countries | n/a |
| Alkhateeb et al. (2016) (15) | ADHD Research in Arab Countries: A Systematic Review of Literature | n/a |
| Wang et al. (2017) (16) | Prevalence of attention deficit/hyperactivity disorder among children and adolescents in China: a systematic review and meta-analysis | Risk of Bias Tool for prevalence studies which was developed by Hoy et al. |
| Li et al. (2018) (17) | [Prevalence of attention deficit and hyperactivity disorder in children in China: a systematic review and Meta-analysis] | cross-sectional study criteria according to STROBE statement |
| Sayal et al. (2018) (18) | ADHD in children and young people: prevalence, care pathways, and service provision | n/a |
| Liu et al. (2018) (19) | The Prevalence of Attention Deficit/Hyperactivity Disorder among Chinese Children and Adolescents | n/a |
| Yadegari et al. (2018) (20) | The Prevalence of Attention Deficient Hyperactivity Disorder Among Iranian Children: A Meta-Analysis | n/a |
| Reale et al. (2018) (21) | ADHD prevalence estimates in Italian children and adolescents: a methodological issue | modified tool developed by Hoy et al. |
| Joseph et al. (2019) (22) | Prevalence of Attention-Deficit Hyperactivity Disorder in India | “Quality assessment checklist for prevalence studies” extracted from Hoy et al. |
| Dobrosavljevic et al. (2020) (23) | Prevalence of attention-deficit/hyperactivity disorder in older adults: A systematic review and meta-analysis | Adjusted Joanna Briggs Institute Critical Appraisal Checklist for Studies Reporting Prevalence Data (Munn et al., 2014) |
| Ayano et al. (2020) (24) | Epidemiology of attention-deficit/hyperactivity disorder (ADHD) in children and adolescents in Africa: a systematic review and meta-analysis | modifed version of the Newcastle–Ottawa Scale (NOS) |
| Hakim Shooshtari et al. (2021) (25) | The prevalence of attention deficit hyperactivity disorder in Iran: An updated systematic review | Quality Assessment Checklist for PrevalenceStudies that assesses sampling, measurement, and analysis method of studies with some questions |
| Cénat et al. (2021) (26) | Prevalence and Risk Factors Associated With Attention-Deficit/Hyperactivity Disorder Among US Black Individuals: A Systematic Review and Meta-analysis | The Joanna Briggs Institute Checklist for Prevalence Studies. |
| Vasileva et al. (2021) (27) | Research review: A meta-analysis of the international prevalence and comorbidity of mental disorders in children between 1 and 7 years | n/a (only publication bias) |
| Song et al. (2021) (28) | The prevalence of adult attention-deficit hyperactivity disorder: A global systematic review and meta-analysis | Strengthening the Reporting of Observational Studies in Epidemiology (STROBE) statement |
| Barican et al. (2022) (29) | Prevalence of childhood mental disorders in high-income countries: a systematic review and meta-analysis to inform policymaking | Adapted risk-of-bias tool developed by Hoy et al |
| Chauhan et al. (2022) (30) | Burden of Attention Deficit Hyperactivity Disorder (ADHD) in Indian Children: A Systematic Review and Meta-Analysis | n/a |
| Caci et al. (2023) (31) | Prevalence rate of ADHD in France: Review of the literature and results from ChiP-ARD study | n/a (according to the authors' comment in the abstract; while most studies are of questionable quality. However, they emphasize that meta-analyses may lose their interpretive value without proper quality assessment.) |
| Ayano et al. (2023) (32) | Prevalence of attention deficit hyperactivity disorder in adults: Umbrella review of evidence generated across the globe | A Measurement Tool to Assess Systematic Reviews (AMSTAR) was used to assess the quality of the included studies (AMSTAR) tool |
| Salari et al. (2023) (33) | The global prevalence of ADHD in children and adolescents: a systematic review and meta-analysis | STROBE checklist |
| Ayano et al. (2023) (34) | The global prevalence of attention deficit hyperactivity disorder in children and adolescents: An umbrella review of meta-analyses | Measurement Tool to Assess Systematic Reviews (AMSTAR) |
| Lynch et al. (2023) (35) | Prevalence of mental health disorders in children and adolescents in the Republic of Ireland: a systematic review | The Joanna Briggs Institute criteria |
| Aljadani et al. (2023) (36) | Prevalence and Risk Factors of Attention Deficit-Hyperactivity Disorder in the Saudi Population: A Systematic Review and Meta-analysis | The National Institutes of Health Quality Assessment Tool for Observational Cohort and Cross-sectional studies |
| Cortese et al. (2023) (37) | Incidence, prevalence, and global burden of ADHD from 1990 to 2019 across 204 countries: data, with critical re-analysis, from the Global Burden of Disease study | n/a |
| Jakobsson et al. (2024) (38) | Meta-Analysis: Prevalence of Youth Mental Disorders in Sub-Saharan Africa | Johanna Briggs Institute Tools for cohort and cross-sectional study designs |
| Mishra et al. (2024) (39) | Prevalence of adult attention deficit hyperactivity disorder in India: a systematic review and a cross-sectional study among young adults in Delhi-NCR | n/a |
| Popit et al. (2024) (40) | Prevalence of attention-deficit hyperactivity disorder (ADHD): systematic review and meta-analysis | n/a |
| Cénat et al. (2024) (41) | Prevalence of ADHD among Black Youth Compared to White, Latino and Asian Youth: A Meta-Analysis | n/a |
| Al-Wardat et al. (2024) (42) | Prevalence of attention-deficit hyperactivity disorder in children, adolescents and adults in the Middle East and North Africa region: a systematic review and meta-analysis | Newcastle-Ottawa Scale (NOS) |
| Girma et al. (2024) (43) | The pooled prevalence of attention-deficit/hyperactivity disorder among children and adolescents in Ethiopia: A systematic review and meta-analysis | The Joanna Briggs Institute (JBI) Critical Appraisal Checklist for cross-sectional study design |
| Sacco et al. (2024) (44) | A systematic review and meta-analysis on the prevalence of mental disorders among children and adolescents in Europe | The Risk of Bias in Prevalence Studies Tool (RBPS) and the Appraisal Tool for Cross-Sectional Studies (AXIS) |
| Ranjan et al. (2024) (45) | Prevalence of attention deficit and hyperactive disorders in South Asian countries: A systematic review and meta-analysis of cross-sectional surveys from 1980 to 2023 | STROBE checklist |
| Azmeraw et al. (2024) (46) | The prevalence of attention-deficit hyperactivity disorder and its associated factors among children in Ethiopia, 2024: a systematic review and meta-analysis | Joanna Briggs Institute (JBI) critical appraisal instrument |

n/a: not applicable

1. Scahill L, Schwab-Stone M. Epidemiology of ADHD in school-age children. Child Adolesc Psychiatr Clin N Am. 2000 Jul;9(3):541–55, vii.

2. Skounti M, Philalithis A, Galanakis E. Variations in prevalence of attention deficit hyperactivity disorder worldwide. Eur J Pediatr. 2007 Feb;166(2):117–23.

3. Polanczyk G, de Lima MS, Horta BL, Biederman J, Rohde LA. The worldwide prevalence of ADHD: a systematic review and metaregression analysis. Am J Psychiatry. 2007 Jun;164(6):942–8.

4. Simon V, Czobor P, Bálint S, Mészáros A, Bitter I. Prevalence and correlates of adult attention-deficit hyperactivity disorder: meta-analysis. Br J Psychiatry. 2009 Mar;194(3):204–11.

5. Farah LG, Fayyad JA, Eapen V, Cassir Y, Salamoun MM, Tabet CC, et al. ADHD in the Arab world: a review of epidemiologic studies. J Atten Disord. 2009 Nov;13(3):211–22.

6. Shooshtary MH, Chimeh N, Najafi M, Mohamadi MR, Yousefi-Nouraie R, Rahimi-Mvaghar A. The prevalence of Attention Deficit Hyperactivity Disorder in Iran: A systematic review. Iran J Psychiatry. 2010;5(3):88–92.

7. Willcutt EG. The prevalence of DSM-IV attention-deficit/hyperactivity disorder: a meta-analytic review. Neurotherapeutics. 2012 Jul;9(3):490–9.

8. Catalá-López F, Peiró S, Ridao M, Sanfélix-Gimeno G, Gènova-Maleras R, Catalá MA. Prevalence of attention deficit hyperactivity disorder among children and adolescents in Spain: a systematic review and meta-analysis of epidemiological studies. BMC Psychiatry. 2012 Oct 12;12:168.

9. Bakare MO. Attention deficit hyperactivity symptoms and disorder (ADHD) among African children: a review of epidemiology and co-morbidities. Afr J Psychiatry (Johannesbg). 2012 Sep;15(5):358–61.

10. Ramos-Quiroga JA, Montoya A, Kutzelnigg A, Deberdt W, Sobanski E. Attention deficit hyperactivity disorder in the European adult population: prevalence, disease awareness, and treatment guidelines. Curr Med Res Opin. 2013 Sep;29(9):1093–104.

11. Polanczyk GV, Willcutt EG, Salum GA, Kieling C, Rohde LA. ADHD prevalence estimates across three decades: an updated systematic review and meta-regression analysis. Int J Epidemiol. 2014 Apr;43(2):434–42.

12. Polanczyk GV, Salum GA, Sugaya LS, Caye A, Rohde LA. Annual research review: A meta-analysis of the worldwide prevalence of mental disorders in children and adolescents. J Child Psychol Psychiatry. 2015 Mar;56(3):345–65.

13. Thomas R, Sanders S, Doust J, Beller E, Glasziou P. Prevalence of attention-deficit/hyperactivity disorder: a systematic review and meta-analysis. Pediatrics. 2015 Apr;135(4):e994-1001.

14. Alhraiwil NJ, Ali A, Househ MS, Al-Shehri AM, El-Metwally AA. Systematic review of the epidemiology of attention deficit hyperactivity disorder in Arab countries. Neurosciences (Riyadh). 2015 Apr;20(2):137–44.

15. Alkhateeb JM, Alhadidi MS. ADHD Research in Arab Countries: A Systematic Review of Literature. J Atten Disord. 2019 Nov 1;23(13):1531–45.

16. Wang T, Liu K, Li Z, Xu Y, Liu Y, Shi W, et al. Prevalence of attention deficit/hyperactivity disorder among children and adolescents in China: a systematic review and meta-analysis. BMC Psychiatry. 2017 19;17(1):32.

17. Li SM, Feng W, Fang F, Dong XH, Zhang ZJ, Yang QQ. [Prevalence of attention deficit and hyperactivity disorder in children in China: a systematic review and Meta-analysis]. Zhonghua Liu Xing Bing Xue Za Zhi. 2018 Jul 10;39(7):993–8.

18. Sayal K, Prasad V, Daley D, Ford T, Coghill D. ADHD in children and young people: prevalence, care pathways, and service provision. Lancet Psychiatry. 2018 Feb;5(2):175–86.

19. Liu A, Xu Y, Yan Q, Tong L. The Prevalence of Attention Deficit/Hyperactivity Disorder among Chinese Children and Adolescents. Sci Rep. 2018 16;8(1):11169.

20. The Prevalence of Attention Deficient Hyperactivity Disorder Among Iranian Children: A Meta-Analysis [Internet]. [cited 2025 May 27]. Available from: https://brieflands.com/articles/ijpbs-8990.html

21. Reale L, Bonati M. ADHD prevalence estimates in Italian children and adolescents: a methodological issue. Ital J Pediatr. 2018 Sep 5;44(1):108.

22. (PDF) Prevalence of attention-deficit hyperactivity disorder in India: A systematic review and meta-analysis. ResearchGate [Internet]. [cited 2025 May 27]; Available from: https://www.researchgate.net/publication/338730743_Prevalence_of_attention-deficit_hyperactivity_disorder_in_India_A_systematic_review_and_meta-analysis

23. Dobrosavljevic M, Solares C, Cortese S, Andershed H, Larsson H. Prevalence of attention-deficit/hyperactivity disorder in older adults: A systematic review and meta-analysis. Neurosci Biobehav Rev. 2020 Nov;118:282–9.

24. Ayano G, Yohannes K, Abraha M. Epidemiology of attention-deficit/hyperactivity disorder (ADHD) in children and adolescents in Africa: a systematic review and meta-analysis. Ann Gen Psychiatry. 2020;19:21.

25. Hakim Shooshtari M, Shariati B, Kamalzadeh L, Naserbakht M, Tayefi B, Taban M. The prevalence of attention deficit hyperactivity disorder in Iran: An updated systematic review. Med J Islam Repub Iran. 2021;35:8.

26. Cénat JM, Blais-Rochette C, Morse C, Vandette MP, Noorishad PG, Kogan C, et al. Prevalence and Risk Factors Associated With Attention-Deficit/Hyperactivity Disorder Among US Black Individuals: A Systematic Review and Meta-analysis. JAMA Psychiatry. 2021 Jan 1;78(1):21–8.

27. Vasileva M, Graf RK, Reinelt T, Petermann U, Petermann F. Research review: A meta-analysis of the international prevalence and comorbidity of mental disorders in children between 1 and 7 years. J Child Psychol Psychiatry. 2021 Apr;62(4):372–81.

28. Song P, Zha M, Yang Q, Zhang Y, Li X, Rudan I. The prevalence of adult attention-deficit hyperactivity disorder: A global systematic review and meta-analysis. J Glob Health. 2021 Feb 11;11:04009.

29. Barican JL, Yung D, Schwartz C, Zheng Y, Georgiades K, Waddell C. Prevalence of childhood mental disorders in high-income countries: a systematic review and meta-analysis to inform policymaking. Evid Based Ment Health. 2022 Feb;25(1):36–44.

30. Chauhan A, Sahu JK, Singh M, Jaiswal N, Agarwal A, Bhanudeep S, et al. Burden of Attention Deficit Hyperactivity Disorder (ADHD) in Indian Children: A Systematic Review and Meta-Analysis. Indian J Pediatr. 2022 Jun;89(6):570–8.

31. Caci H. Prevalence rate of ADHD in France: Review of the literature and results from ChiP-ARD study. Encephale. 2023 Dec;49(6):624–31.

32. Ayano G, Tsegay L, Gizachew Y, Necho M, Yohannes K, Abraha M, et al. Prevalence of attention deficit hyperactivity disorder in adults: Umbrella review of evidence generated across the globe. Psychiatry Res. 2023 Oct;328:115449.

33. Salari N, Ghasemi H, Abdoli N, Rahmani A, Shiri MH, Hashemian AH, et al. The global prevalence of ADHD in children and adolescents: a systematic review and meta-analysis. Ital J Pediatr. 2023 Apr 20;49:48.

34. Ayano G, Demelash S, Gizachew Y, Tsegay L, Alati R. The global prevalence of attention deficit hyperactivity disorder in children and adolescents: An umbrella review of meta-analyses. Journal of Affective Disorders. 2023 Oct 15;339:860–6.

35. Lynch S, McDonnell T, Leahy D, Gavin B, McNicholas F. Prevalence of mental health disorders in children and adolescents in the Republic of Ireland: a systematic review. Ir J Psychol Med. 2023 Mar;40(1):51–62.

36. Aljadani AH, Alshammari TS, Sadaqir RI, Alrashede NOE, Aldajani BM, Almehmadi SA, et al. Prevalence and Risk Factors of Attention Deficit-Hyperactivity Disorder in the Saudi Population: A Systematic Review and Meta-analysis. Saudi J Med Med Sci. 2023 Jun;11(2):126–34.

37. Cortese S, Song M, Farhat LC, Yon DK, Lee SW, Kim MS, et al. Incidence, prevalence, and global burden of ADHD from 1990 to 2019 across 204 countries: data, with critical re-analysis, from the Global Burden of Disease study. Mol Psychiatry. 2023 Nov;28(11):4823–30.

38. Jakobsson CE, Johnson NE, Ochuku B, Baseke R, Wong E, Musyimi CW, et al. Meta-Analysis: Prevalence of Youth Mental Disorders in Sub-Saharan Africa. Glob Ment Health (Camb). 2024;11:e109.

39. Mishra S, Chaudhary V, Saraswathy KN, Shekhawat LS, Devi NK. Prevalence of adult attention deficit hyperactivity disorder in India: a systematic review and a cross-sectional study among young adults in Delhi-NCR. Soc Psychiatry Psychiatr Epidemiol. 2025 Apr;60(4):785–96.

40. Popit S, Serod K, Locatelli I, Stuhec M. Prevalence of attention-deficit hyperactivity disorder (ADHD): systematic review and meta-analysis. Eur Psychiatry. 2024 Oct 9;67(1):e68.

41. Cénat JM, Kokou-Kpolou CK, Blais-Rochette C, Morse C, Vandette MP, Dalexis RD, et al. Prevalence of ADHD among Black Youth Compared to White, Latino and Asian Youth: A Meta-Analysis. J Clin Child Adolesc Psychol. 2022 Apr 15;1–16.

42. Al-Wardat M, Etoom M, Almhdawi KA, Hawamdeh Z, Khader Y. Prevalence of attention-deficit hyperactivity disorder in children, adolescents and adults in the Middle East and North Africa region: a systematic review and meta-analysis. BMJ Open. 2024 Jan 18;14(1):e078849.

43. Girma D, Abita Z, Adugna A, Alie MS, Shifera N, Abebe GF. The pooled prevalence of attention-deficit/hyperactivity disorder among children and adolescents in Ethiopia: A systematic review and meta-analysis. PLoS One. 2024;19(7):e0307173.

44. Sacco R, Camilleri N, Eberhardt J, Umla-Runge K, Newbury-Birch D. A systematic review and meta-analysis on the prevalence of mental disorders among children and adolescents in Europe. Eur Child Adolesc Psychiatry. 2024 Sep;33(9):2877–94.

45. Ranjan JK, Kumari R, Choudhary A. Prevalence of attention deficit and hyperactive disorders in South Asian countries: A systematic review and meta-analysis of cross-sectional surveys from 1980 to 2023. Asian J Psychiatr. 2024 Apr;94:103970.

46. Azmeraw M, Temesgen D, Kassaw A, Zemariam AB, Kerebeh G, Abebe GK, et al. The prevalence of attention-deficit hyperactivity disorder and its associated factors among children in Ethiopia, 2024: a systematic review and meta-analysis. Front Child Adolesc Psychiatry. 2024;3:1425841.
